# Current Understanding of the Emerging Role of Prolidase in Cellular Metabolism

**DOI:** 10.3390/ijms21165906

**Published:** 2020-08-17

**Authors:** Magdalena Misiura, Wojciech Miltyk

**Affiliations:** Department of Analysis and Bioanalysis of Medicines, Medical University of Bialystok, 15-089 Białystok, Poland; magdalena.misiura@umb.edu.pl

**Keywords:** prolidase, PEPD, EGFR, cellular metabolism

## Abstract

Prolidase [EC 3.4.13.9], known as PEPD, cleaves di- and tripeptides containing carboxyl-terminal proline or hydroxyproline. For decades, prolidase has been thoroughly investigated, and several mechanisms regulating its activity are known, including the activation of the β_1_-integrin receptor, insulin-like growth factor 1 receptor (IGF-1) receptor, and transforming growth factor (TGF)-β_1_ receptor. This process may result in increased availability of proline in the mitochondrial proline cycle, thus making proline serve as a substrate for the resynthesis of collagen, an intracellular signaling molecule. However, as a ligand, PEPD can bind directly to the epidermal growth factor receptor (EGFR, epidermal growth factor receptor 2 (HER2)) and regulate cellular metabolism. Recent reports have indicated that PEPD protects p53 from uncontrolled p53 subcellular activation and its translocation between cellular compartments. PEPD also participates in the maturation of the interferon α/β receptor by regulating its expression. In addition to the biological effects, prolidase demonstrates clinical significance reflected in the disease known as prolidase deficiency. It is also known that prolidase activity is affected in collagen metabolism disorders, metabolic, and oncological conditions. In this article, we review the latest knowledge about prolidase and highlight its biological function, and thus provide an in-depth understanding of prolidase as a dipeptidase and protein regulating the function of key biomolecules in cellular metabolism.

## 1. Introduction

Proline has a unique pyrrolidine ring that protects the polypeptide structure from hydrolysis. The presence of proline in numerous biomolecules (e.g., neuroactive peptides or growth factors) prevents unexpected proteolysis in order to maintain their biological activity [1]. However, there are factors responsible for the degradation of peptides containing proline at the C-terminus. One of the enzymes involved in this process is prolidase [EC 3.4.13.9], which catalyzes the hydrolysis of X-Pro or X-Hyp to proline or hydroxyproline and X amino acid [2]. Prolidase (PEPD) belongs to the group of dipeptidases and cleaves di- and tripeptide containing carboxyl-terminal proline or hydroxyproline. The most specific substrate for this enzyme activity is glycyl-proline (Gly-Pro) [2]. Its enzymatic properties link to the disease known as prolidase deficiency (PD), which is manifested by massive imidodipeptiduria, hard-to-heal wounds, mental retardation, and impaired immune system. To date, no effective PD treatment has been developed [3]. Moreover, there are reports indicating the clinical relevance of prolidase in collagen metabolism malfunctions [4,5,6,7,8,9,10,11,12,13], metabolic [14,15,16,17,18,19,20], and oncological disorders [21,22,23,24,25]. In addition to its catalytic activity, prolidase regulates numerous biological processes. At the cellular level, PEPD acts as a regulator of epidermal growth factor receptor (EGFR) and epidermal growth factor receptor 2 (HER2)-dependent signaling pathways [26,27,28,29,30], p53 activity [31], and expression of the interferon α/β receptor [32].

The purpose of this review is to present the latest knowledge about prolidase as well as its biological significance at the cellular level in the aspect of its catalytic-dependent and -independent biological activity. The enzymatic-dependent function of prolidase concerning its clinical importance in PD, collagen turnover, metabolic conditions, and cancers is discussed. We focus on in-depth understanding of the biological properties of prolidase as a dipeptidase and a molecule regulating the function of key biofactors in the cellular metabolism.

## 2. Regulatory Functions of Prolidase

**Figure 1:**
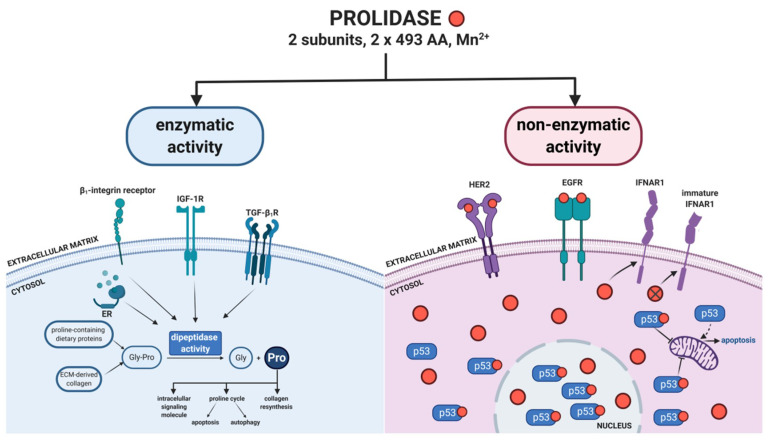
Enzymatic and non-enzymatic prolidase activity. Prolidase exhibits dual mechanism of biological activity. As an enzyme, prolidase provides proline for collagen resynthesis. The amino acid acts as a signaling molecule and a mediator in mitochondrial proline cycle. Extracellularly, prolidase binds directly to EGFR and HER2, while intracellularly it regulates the function of p53 and IFNAR1. Red dots indicate prolidase. ER—estrogen receptor, IGF-1R—insulin-like growth factor 1 receptor, HER2—epidermal growth factor receptor 2, EGFR—epidermal growth factor receptor, IFNAR1—interferon α/β receptor, Pro—proline, Gly—glycine, Gly-Pro—glycyl-proline, TGF-β_1_R—transforming growth factor β_1_ receptor. Created with BioRender.com.

In the 1950s, Adams et al. [33] published the first report on prolidase. For the next decades, researchers focused solely on the enzymatic function of this enzyme. Most publications concern prolidase deficiency—a genetic disease resulted from a decrease in or lack of PEPD activity. Disturbed prolidase activity has been reported in various pathological conditions associated with collagen metabolism and tumors. A breakthrough in the research on the biological role of prolidase was the study conducted by Yang et al. [26], presenting a new unknown function of PEPD as an epidermal growth factor receptor ligand. Since then, knowledge about the biological role of PEPD catalytic-independent activity has been expanded. Apart from the said finding, the role of prolidase in regulating p53 and interferon α/β receptor has also been discovered. The paragraphs below in this section describe novel functions of PEPD as a cellular regulator. Figure 1 presents biological activity of prolidase as an enzyme as well as a regulatory protein in cellular metabolism.

Enzyme-independent biological activity of PEPD includes its role in regulating the functions of other molecules. Recent scientific reports have expanded the knowledge of prolidase and its interactions with biomolecules at the cellular level. Researchers have demonstrated the role of prolidase as an EGFR and HER2 ligand regulating signaling pathways dependent on these receptors. PEPD also regulates the function of p53 and plasma serine proteases as well as expression of interferon α/β receptor.

### 2.1. Prolidase as an Epidermal Growth Factor Receptor (ErbB1/EGFR) Ligand

Yang et al. shed new light on the function of prolidase by publishing several papers [26,27,28,29,30] in which the authors demonstrated that PEPD is a ligand of receptors belonging to the family of epidermal growth factor receptors (ErbB1/EGFR and ErbB2/HER2). They showed that the affinity of prolidase to these receptors is lower than EGF, but the effects of EGFR-dependent signal induction last longer. The activation of these signaling pathways does not require any enzymatic activity of prolidase, which suggests the new role of PEPD in cellular metabolism. Structurally, EGFR is a transmembrane receptor comprised of: the intracellular region at the carboxyl terminus, exhibiting protein kinase activity and the extracellular region that binds to a ligand. In addition to PEPD, several EGFR ligands have been identified, e.g., heparin-binding EGF-like growth factor (HB-EGF), transforming growth factor (TGF), amphiregulin, epiregulin, and neuregulin [34]. However, prolidase, as a homodimeric molecule, differs structurally from the group of EGFR ligands. PEPD does not share the characteristic EGF motif with other ligands (CX_7_CX_4–5_CX_10–13_CXCX_8_GXRC—X represents an amino acid) and its cytoplasmic location differs from that of typical EGFR ligands [35]. Still, it is not known which domain or region of PEPD binds to the extracellular domain of EGFR. Binding of prolidase to the EGFR extracellular domain causes its dimerization. As a result, the intracellular domain with protein kinase activity conducts a signal to downstream proteins in the phosphoinositide 3-kinase (PI3K)/protein kinase B (Akt)/mammalian target of rapamycin (mTOR), Ras/Raf/extracellular signal-regulated kinase (ERK), and Janus kinase (JAK)/signal transducer and activator of transcription 3 (STAT3) pathways. Activated proteins stimulate transcription of genes associated with cell growth, differentiation, and proliferation [36].

**Figure 2:**
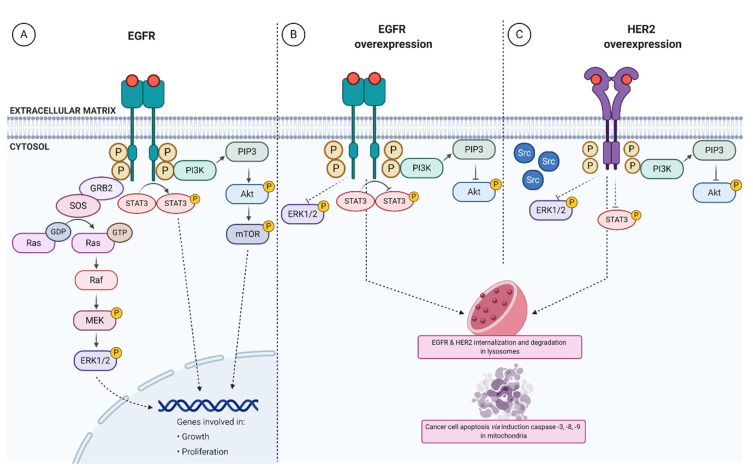
Prolidase-dependent EGFR- and HER2-downstream signaling. Prolidase binds to EGFR and HER2, evoking intracellular responses. (**A**) Under physiological conditions, direct binding of PEPD to EGF receptor results in the induction of pro-growth and pro-proliferation pathways such as phosphoinositide 3-kinase (PI3K)/protein kinase B (Akt)/mammalian target of rapamycin (mTOR), extracellular signal-regulated kinase (ERK)1/2, and signal transducer and activator of transcription 3 (STAT3). (**B**) Under overexpression of EGFR, prolidase silences Akt, ERK1/2, and STAT3 pathways followed by internalization and degradation of the receptor. (**C**) PEPD affects upregulated HER2 via dissociation of HER2-Src complex, inhibition of Akt, ERK1/2 and STAT3 pathways, and induction of apoptosis. Red dots indicate prolidase and circled ‘P’ presents phosphorylation event. HER2—epidermal growth factor receptor 2, EGFR—epidermal growth factor receptor. Created with BioRender.com.

The first Yang’s discovery [26] proved that prolidase binds directly to EGFR and activates the receptor in a dose-dependent manner. Comparing the affinity of EGF and PEPD to the EGFR extracellular domain, EGF is a more potent ligand than prolidase. In the study of the aforementioned author, the dissociation constant (K_d_) was around 15nM for EGF, while K_d_ for PEPD established at 5.3 µM, which indicated that EGF is an about 350 times stronger ligand for EGFR compared to PEPD. Further studies confirmed the affinity of PEPD to EGFR with K_d_ equaling 17.7 nM [29]. It is probable that these discrepancies in K_d_ resulted from differences in the experimental model. In the first research [26], the model used for the study was the EGFR-Fc immunoglobulin G (IgG) 1 chimera, while the second model [29] to assess K_d_ of PEPD-EGFR binding represented the full-length EGF receptor. It is known that the extracellular fragment of EGFR comprises four domains. Prolidase binds to domain 2 as opposed to EGF, which binds to domains 1 and 3 of EGFR. Prolidase is associated with EGFR only on the cell surface as a homodimer, eventually forming a tetrameter (EGFR dimer + PEPD dimer) [29]. An EGF, as a stronger EGFR ligand, displaces PEPD from its bond with EGFR. Confocal images demonstrated that PEPD and EGFR colocalize the cell membrane, which supports the hypothesis that PEPD binds to EGFR in the form of a ligand-receptor relationship. At low concentration, prolidase (2.7 nM) can activate EGFR by phosphorylation of tyrosine at positions 1068 and 1173 followed by EGFR-downstream protein induction (Akt, STAT3, and ERK1/2). Figure 2A demonstrates PEPD-dependent EGFR-downstream signaling pathways. This finding shows that PEPD stimulates three different downstream signaling pathways of EGFR. The effects of equal EGF and PEPD concentrations on the aforementioned pathways are similar. EGF activates EGFR-dependent signaling faster than PEPD, while prolidase-dependent stimulation lasts longer. The most likely explanation for this phenomenon is that EGFR is internalized and degraded more slowly under PEPD treatment. Applying EGFR inhibitor resulted in a nearly complete blockade of ERK1/2 phosphorylation upon PEPD treatment. EGFR-downstream signaling pathways lead to increased DNA synthesis in a dose-dependent manner upon prolidase stimulation. These findings appear promising for the improvement of regenerative therapy, aiming to promote cell proliferation and growth. Through EGFR-dependent stimulation, PEPD may be a beneficial factor in the treatment of diseases manifested by ulceration or chronic inflammation [37].

Yang et al. [26] discovered that the prolidase enzymatic activity is not required to activate the EGF receptor and its downstream signaling proteins. The effect of active and enzymatically inactive forms of prolidase confirms this statement. The phosphorylation level of EGFR and downstream kinases is comparable between wild-type PEPD and its enzymatically inactive mutant (PEPD^G278D^). However, intracellular PEPD does not activate EGFR—the molecule has to be present in the extracellular space. Therefore, a question arises of the source of prolidase stimulating this receptor. The most likely sources of PEPD are damaged cells that release cellular content, including cytosolic prolidase. The in vivo experiments showed significantly increased PEPD concentration in the bloodstream after chemical damage to liver cells. PEPD concentration reached 3 nM compared to the control group in which it did not exceed 1 nM. It is known that 2.7 nM of PEPD is sufficient to stimulate EGFR [26]. Under pathological conditions, PEPD level is higher than that concentration, which leads to another question: does prolidase undergo extracellular degradation preventing it from unexpected EGFR stimulation? The recent paper has revealed that serine proteases can inactivate prolidase in plasma via intrinsic and extrinsic cascades of coagulation. The study has demonstrated that factor XII initiates PEPD proteolysis by activating factor X and factor II, which stimulate factor VII. Activated factor VII (FVIIa) degrades prolidase *in vivo*. FVIIa exhibits trypsin-like serine protease activity by cleaving peptides containing C-terminal arginine or lysine. The authors showed that FVIIa inactivated prolidase. However, the mechanism of PEPD proteolysis remains unexplained [38].

The study reporting PEPD-dependent stimulation of EGFR-downstream signaling pathways contributed to further studies on PEPD-related effects under pathological conditions. EGF receptor overexpression is observed in numerous cancers, including breast, lung, colon cancer, and squamous cell carcinoma [39]; therefore, an attempt was made to assess the effect of prolidase on cell metabolism in conditions of EGFR overexpression. The authors discovered that the enzymatically inactive PEPD^G278D^ silences EGFR-downstream signaling pathways, inhibiting tumor cell proliferation and growth in vivo. Tumor cells in EGFR overexpression were more prone to prolidase, which resulted in inhibition of EGFR phosphorylation and downstream proteins such as Akt and ERK1/2, as well as STAT3 signaling [29]. Figure 2B presents PEPD-related inhibitory effect on overexpressed EGF receptor and EGFR-dependent pathways. The mechanism of EGFR activation by PEPD has not yet been thoroughly studied. It is unknown whether the PEPD-EGFR bond affects cell metabolism in an autocrine or paracrine manner. PEPD is likely to act as an autocrine factor on cells, but there is no evidence for its paracrine effect. Another unclear matter that requires explanation is: which PEPD domain binds to EGFR. The mechanism of prolidase deficiency (PD) is also noteworthy. So far, the most probable cause for PD is diminished enzymatic activity of prolidase. However, considering the newly discovered function of PEPD, a probable PD mechanism might be the underlying excessive release of PEPD from damaged cells and its interactions with other biomolecules including EGFR. It is possible that prolidase concentration in the bloodstream is not reduced in patients with PD. However, there are no papers supporting this hypothesis, and further research explaining the mechanism of PD is crucial. Until now, PD remains incurable, and understanding the causes of the disease may contribute to the development of an effective therapeutic strategy.

PEPD binding to EGFR may be a potential target in treatment of cancers with EGFR overexpression. Since the enzymatically inactive prolidase (PEPD^G278D^) inhibits pro-proliferative signals, it offers a new promising strategy for oncological therapy.

### 2.2. Prolidase as an ErbB2/HER2 Ligand

Yang et al. [28] found that PEPD also binds to HER2 which, unlike EGFR, is comprised of four domains in the extracellular region. PEPD binds to domain 3, thus contributing to the dimerization of this receptor. It has been demonstrated that PEPD does not bind to the HER2 transmembrane domain or intracellular region. The affinity of prolidase for this receptor was estimated at K_d_ = 7.3 nM at which prolidase specifically binds to the receptor. HER2 phosphorylation is induced by PEPD, gradually saturating the receptor and leading to its dimerization. HER2, similarly to EGFR, is activated independently of the enzymatic function of prolidase. By using enzymatically inactive prolidase, it has been proven that stimulation of this receptor remains unchanged. Furthermore, intracellular prolidase does not affect HER2-downstream pathways. Overexpressed PEPD entails a significant elevation of its concentration, so PEPD appears to be secreted from the cells. According to the presented results, PEPD concentration in the medium did not exceed 0.3 nM; hence, it could not affect the function of the receptor. No mechanism has yet been found to explain how the enzyme is released from normal cells. PEPD silences HER2-downstream signals under HER2 overexpression, which is another similarity with EGF receptor. The mechanism underlying the inhibition of HER2-downstream signaling under PEPD treatment is based on disruption of HER2-Src association. As a result, DNA synthesis and cell proliferation, invasion, and migration are strongly hindered. This finding indicates a new therapeutic strategy in the treatment of HER-positive carcinomas. However, some doubts occur because HER2 function depends on other receptors from this family. For example, ErbB3 can stimulate HER2 dimerization [27]. To explain this phenomenon, the effect of prolidase on tumor progression with coexisting HER2 overexpression was assessed [28]. Intraperitoneal administration of PEPD at a dose of 0.2 mg/kg has been shown to inhibit tumor growth in vivo only with the concomitant overexpression of HER2. The authors proposed the use of a combination of prolidase and enoxaparin (EP) as a therapeutic option. EP (as low-molecular-weight heparin) increases plasma prolidase levels by inhibiting PEPD proteolysis [38]. EP itself does not inhibit tumor growth; however, it lowers the dose of prolidase while its plasma concentration is sufficient. As a result, prolidase suppresses HER2-dependent intracellular signals by inhibiting the phosphorylation of Src, Akt, STAT3, and ERK1/2. The effect of PEPD-HER2 interaction is internalization and subsequent HER2 degradation as presented in Figure 2C. Although PEPD does not bind to ErbB3, prolidase prevents this receptor from phosphorylation by inhibiting Akt expression. It is known that HER2 overexpression is accompanied by phosphorylated Akt, which affects ErbB3 dimerization. In addition, PEPD promotes apoptosis in tumor tissues through decreased expression of B-cell lymphoma 2 (BCL-2) and Bcl-2-associated X (BAX) as well as upregulated expression of caspase-3, -8, -9. The effect of PEPD on HER2-overexpressed signaling in tumors is the suppression of pro-proliferative signaling pathways, induction of apoptosis, and inhibition of tumor progression. In vivo model indicates no signs of toxicity, and body weight of mice remains unchanged [28]. After PEPD administration, it has been observed that the enzymatically inactive PEPD^G278D^ mutant has a stronger inhibitory effect on tumor weight than its enzymatically active form. The reason for PEPD being a limiting factor for tumor mass weight remains to be explored. Based on these observations, further experiments were performed to evaluate the therapeutic effect of PEPD^G278D^ on drug-resistant HER2-positive breast cancer [30]. The enzymatically inactive mutant exhibits cancer in two-step process: in the first phase, it disturbs the interaction of HER2 with other receptors in this family (EGFR, ErbB3) as well as other tyrosine kinase receptors (MET, IGF-1R). It is likely that PEPD disconnects the bond between HER2 and mucin 4 (marker present in 60% of HER2-positive breast cancers, probably related to drug resistance). PEPD^G278D^ also interferes with the HER2-downstream signaling. In the second phase, the receptor undergoes slow internalization followed by degradation in lysosomes. Another beneficial anti-tumor effect of PEPD is related to increased cancer cell sensitivity to drug treatment. Moreover, it was observed that drug-resistant HER2-positive breast cancer cells are more sensitive to the enzymatically inactive PEPD^G278D^ mutant, giving basis for the development of new therapeutic options for this group of cancers.

### 2.3. Prolidase as a p53 Activity Regulator

In recent years, a new function of prolidase in p53 function has been discovered. PEPD is a key regulator of the key tumor suppressor protein [31]. The report reveals an important role in controlling cellular functions associated with the cell cycle, DNA repair, apoptosis, and cellular metabolism. p53, as the guardian of the genome, protects cells from uncontrolled cell division that may lead to proliferation of mutated cells and promote tumor progression [40]. There are several upstream and downstream regulating mechanisms of p53 function such as its post-translational modifications mediated by mutated in ataxia telangiectasia (ATM), ATM and RAD3-related (ATR), p38 MAP kinase (MAPK), ERK1/2, Checkpoint kinase 1 (CHK1), Checkpoint kinase 2 (CHK2), interactions with murine double minute 2 (MDM2), murine double minute 4 (MDM4), wild-type p53-induced phosphatase (WIP1), p21 as well as chromatin. Apart from these, p53 dynamics itself affects its biological status [41]. The regulation of the guardian of the genome is complex and still needs in-depth investigation. Among those mechanisms, prolidase also exhibits regulatory function on p53 through (1) limiting p53 subcellular transport and (2) inhibiting p53 phosphorylation [31].

The absence of prolidase limits cell survival, as confirmed by *PEPD* gene silencing. It has been observed that p53 activation is associated with PEPD silencing. Further analysis indicates that p53 is located in the cytosol, nucleus, and mitochondria, whereas prolidase occurs only in the cytosol and nucleus. It is the very first evidence to support the assumption that prolidase can translocate to the nucleus [42]. PEPD prevents p53 from its translocation into mitochondria where apoptosis is initiated. Prolidase can also inhibit p53 transcriptional activity by inhibiting the protein phosphorylation in its transactivation domain. Under silenced PEPD conditions, p53 phosphorylation at the Ser6 and Ser15 positions is promoted. The aforementioned findings prove that prolidase regulates both transcription-dependent and -independent functions of p53 [31].

Yang et al. [31] demonstrated that PEPD regulates the activity of p53 through direct binding to this protein. They showed that PEPD catalytic domain is bound to the proline-rich domain of p53; however, PEPD motif binding to p53 is unknown. Prolidase enzymatic activity is not required for either regulating p53 function or binding to this transcription factor. About 6% of prolidase molecules have been found to bind to p53, while more than half of p53 molecules bind to PEPD, which indicated that prolidase protects p53 from uncontrolled activation. Under cellular stress, the PEPD-p53 complex dissociates, releasing and activating p53. An example of stress conditions at the cellular level is oxidative stress caused by reactive oxygen species (ROS). Restoration of redox balance prevents PEPD-p53 from dissociation as well as subsequent inhibition of cell growth and induction of apoptosis. Experimentally, oxidative balance was restored by N-acetylcysteine as a ‘scavenger’ of ROS. As a result, p53 activity was diminished via complexing by PEPD [31]. Since oxidative stress is generated in patients undergoing chemotherapy, PEPD may be a useful factor in combination with chemotherapeutics, although further research is required in this field. So far, opinions of experts on the administration of anti-cancer drugs accompanied by antioxidants are varied [43,44].

In summary, the report illustrates the important biological function of PEPD, independently of its catalytic activity, in regulating p53 activity. The PEPD-p53 complex has been observed in the nucleus and cytosol. The dual mechanism of p53 regulation by prolidase includes inhibition of subcellular translocation and p53 phosphorylation. Disrupted redox balance leads to dissociation of the complex and stimulation of p53 activity. Since p53 determines the cell fate, this finding may contribute to further research on the pathomechanisms of numerous diseases accompanied by oxidative stress.

### 2.4. Prolidase as a Regulator of Interferon α/β Receptor

Lubick et al. [32] presented a new physiological function of prolidase in which PEPD modulates the functionality of interferon α/β receptor (IFNAR1). Interferon α/β-dependent signaling is a key pathway involved in the immune response against viruses, i.e., tick-borne encephalitis virus and West Nile virus. The research results indicate that IFNAR1 expression is diminished during a flavivirus-induced infection. A common feature of the flaviviruses is the use of non-structural protein 5 (NS5) as an IFNAR1 antagonist [45,46], leading to the suppression of the immune response. The research shows that the N-terminal PEPD domain binds to NS5, decreasing interferon α/β receptor expression [32]. To clarify the role of prolidase in regulating IFNAR1 expression, the authors silenced PEPD and observed that post-translational modification of the receptor is impaired. The glycosylation is required for IFNAR1 to perform receptor functions. The conclusion is that prolidase is involved in the maturation of interferon α/β receptor. Similarly, to other non-enzymatic properties of prolidase, PEPD regulates IFNAR1 function independently of its catalytic activity [32]. To sum up, prolidase—as a cytosolic molecule—is blocked by viral protein, which leads to inhibition of IFNAR1-dependent immune response and more precisely: the role of PEPD in the immune response against flaviviruses could initiate the development of PEPD-based antiviral therapies.

## 3. Enzyme-Dependent Activity of Prolidase

At the cellular level, the biological processes dependent on the enzymatic activity of prolidase result from the biological activity of enzymatic reaction products: proline or hydroxyproline (Pro or Hyp, respectively). Most scientific reports focus on biological properties of proline. Enzymatic activity of PEPD is necessary for the collagen turnover as the main component of the extracellular matrix, participating in the proteolysis of di- and tripeptide derived from degradation of collagen and proline-containing proteins.

### 3.1. Prolidase as a Dipeptidase: General Structure, Physical Properties, and Substrate Specificity

Prolidase belongs to the family of metallopeptidases dependent on divalent cations that enable its catalytic activity. PEPD is known as X-Pro dipeptidase, proline dipeptidase, imidodipeptidase, and peptidase D [1].

From a molecular point of view, prolidase is encoded by the *PEPD* gene located on the long arm of chromosome 19 at locus 13.11. It has been observed that the gene structure has 15 exons [2]. Point mutations in this gene are responsible for the lack or reduction of the enzymatic activity, and thus causing prolidase deficiency. It is known that the *PEPD* gene has 29 point mutations that result in a reduction or complete loss of the enzymatic activity. Out of these, eight point mutations are of clinical significance [47]. Prolidase can exist in three isoforms depending on the transcriptomic variant. Isoform 1 is the product of the longest transcript, while prolidase isoform 2 is shortened by an internal segment from 184 to 224 nucleotide. Because of alternative splicing, isoform 3 is deprived of a nucleotide fragment from 68 to 131 nucleotides [48]. It is known that in eukaryotes, prolidase undergoes post-translational modifications such as glycosylation and phosphorylation. The analysis of the carbohydrate content in the prolidase structure showed that it constitutes 0.5% of the total protein mass [49]. This report presents prolidase as a glycoprotein; however, further research is needed to assess the binding sites of carbohydrate groups and evaluate whether glycosylation affects biological properties of prolidase. Indirectly, it has been demonstrated that glycosylation does not influence the catalytic activity of the enzyme [42]. Another study showed that N-glycosylation can occur at N13 and N172 sites, and O-glycosylation—at position T458 in the amino acid chain [50]. In terms of PEPD phosphorylation, there have been several reports [51,52] confirming that this post-translational modification increases PEPD enzymatic activity. The sites of phosphorylation include Ser109, Ser134, Ser198, Ser236, Thr86, Tyr117, and Tyr124. The authors showed that these amino acids are phosphorylated as a response to stimulation via NO/cGMP/MAPK pathways, which means that nitric oxide regulates prolidase activity. Ysrayl et al. provided further evidence supporting the phenomenon of prolidase phosphorylation [53]. They observed that cocaine stimulates prolidase phosphorylation and increases its enzymatic activity. They also demonstrated that phosphorylation of prolidase depends on the iNOS pathway, which inhibits the level of phosphorylated protein, which is consistent with the previous study [51].

Structurally, human prolidase is a homodimer consisting of two subunits, 493 amino acids (AA) each [54]. The molecular weight of one subunit is 58 kDa [42]. Both subunits are comprised of the N- and C-terminal domains. The carboxyl-terminal domain (185–493 AA) shares the structure with peptidases from the ‘pita-bread’ family (e.g., aminopeptidase P, methionine aminopeptidase, and creatinase) [55]. In this domain, there is an active center in which the Mn^2+^ ion is necessary for its enzymatic activity. The substrate (Gly-Pro) binds to the active center in the C-terminal domain, while the N-terminal domain (1–185 AA) remains less closely linked to the substrate. The disulphide bond between Cys158A and Cys158B links the monomers. Notably, this disulphide bridge is only present in the inactive enzyme-substrate complex [56]. The divalent cations: Zn^2+^, Mg^2+^, Ca^2+^, Co^2+^ can be also present as cofactors required for PEPD enzymatic activity. However, prolidase activity is decreased if one of these cations is located in the active center. Under these conditions, the activity of prolidase drops below 30% [20]. Interestingly, in *Saccharomyces cerevisiae*, Cu^2+^ and Zn^2+^ strongly inhibit prolidase activity [57]. Lupi et al. [42] compared the catalytic activity of endogenous and recombinant prolidase and discovered that optimal conditions for their maximum catalytic activity are: pH 7.8 and 37 °C or 50 °C, respectively.

Prolidase belongs to the group of hydrolases; therefore, the enzymatic reaction catalyzed by PEPD requires H_2_O. Wilk et al. [54] established the mechanism of the reaction catalyzed by recombinant human prolidase. First, water binds to Mn^2+^ ions in the active center of prolidase, which leads to the formation of a hydroxyl ion. Then, Gly-Pro binds to the active center, resulting in a change of the enzyme conformation. Approaching His255, it coordinates the -COOH group in the substrate. The =O and -NH_2_ groups in Gly-Pro are stabilized by manganese ions, providing a positive charge at the carbon atom. A nucleophilic hydroxyl ion attack is followed by a break in the peptide bond, and the products (Gly, Pro) leave the active center. First, Gly is released followed by proline, and then the enzyme returns to its original conformation. The last step involves replenishing water and restoring the enzymatic activity of prolidase.

PEPD exhibits the highest specificity for Gly-Pro in the *trans* conformation [58]. Although prolidase has the highest catalytic activity against Gly-Pro [42], it also hydrolyses other C-terminal proline-containing dipeptides such as Ala-Pro, Phe-Pro, Met-Pro, Val-Pro, and Leu-Pro [50]. Unlike many proteases, prolidase is present in the cytosol [42]. However, it is not known why prolidase occurs in that subcellular location. PEPD is abundantly expressed in enterocytes, where it is probably involved in the hydrolysis of dietary proline-containing dipeptides [59]. Regarding the tissue specificity of prolidase, the highest level of prolidase mRNA expression is observed in the kidneys, small intestine, and duodenum [60], while high prolidase activity has been reported in erythrocytes and human skin fibroblasts [50]. Guszczyn et al. [61] showed that platelet-rich plasma is an important source of prolidase. Apart from mammalian tissues, prolidase also occurs in numerous bacterial species (*Pyrococcus furiosus, Pyrococcus horikoshii, Alteromonas sp., Lactobacillus casei, Lactococcus lactis*) [62].

The key source of substrate (Gly-Pro) are proteins rich in amino acid sequences containing C-terminal proline or hydroxyproline, e.g., collagen [63], complement component C1q [64], dietary proteins [65], and many biomolecules such as substance P, plasminogen, oxytocin, vasopressin, and angiotensin [28]. Out of these, collagen is the most abundant source of Gly-Pro. Its molecule comprises three polypeptide chains in which the Gly-X-Y triplet is commonly repeated. Mostly X and Y are occupied by proline and hydroxyproline, respectively [66]. Thus, a significant role of PEPD is reflected in extracellular matrix (ECM) remodeling as collagen constitutes its structural protein [67,68]. Collagen degradation is initiated by metalloproteinases [69]. Then, the proteolysis of collagen breakdown products by cathepsins and peptidases takes place in lysosomes. However, they cannot degrade di- and tripeptides containing C-terminal proline or hydroxyproline. Prolidase acting in the cytosol releases free amino acids from dipeptides [70]. This process is impaired in prolidase deficiency, manifested by skin lesions resulting from disturbed collagen metabolism. Another symptom of PD is immunodeficiency, which is probably a consequence of impairment in the C1q complement component built from Gly-Pro repeats [64]. Prolidase also plays a role in the deactivation of C-terminal proline-containing neuropeptides, which could be related to mental disorders in patients with PD. Hui et al. [71] observed increased enzymatic activity in the brain tissue. In the central nervous system (CNS), prolidase is responsible for proline delivery. Out of symptoms of prolidase deficiency, mental retardation may occur because of a low level of proline as a neurotransmitter. However, there have been reports in which an increase of proline in CNS results in increased glutamate concentration, leading to neuronal death due to excessive stimulation of N-methyl-D-aspartate (NMDA) receptors. This mechanism could explain the relationship between increased prolidase activity and the pathogenesis of some neurological disorders [72].

### 3.2. Biological Significance of Prolidase as a Dipeptidase

At the cellular level, prolidase activity is regulated by several mechanisms. Stimulation of the β_1_-integrin receptor by type I collagen [30], leading to autophosphorylation of FAK kinase capable of interacting with Grb2 and Src, induces prolidase activity. The signal is further transmitted through SoS, Ras, and Raf pathway to the ERK1/2 kinases. The signaling results in transcription of genes involved in cell growth regulation as well as proliferation [73]. An increase in prolidase activity was also observed while assessing the effect of thrombin as a β_1_-integrin receptor agonist. The interaction of thrombin with this receptor stimulates MAP kinase pathway. In relation to intensified PEPD activity, an increase in collagen biosynthesis has been observed, which confirms that stimulation of β_1_-integrin receptor regulates the availability of proline used as a substrate for collagen synthesis [74]. Prolidase activity is also regulated by an IGF-1 receptor (IGF-1R)-dependent pathway [75], which induces pathways stimulating cell growth, proliferation, and collagen biosynthesis. Thus, prolidase can directly limit collagen biosynthesis at both the transcriptional [68,76] and post-transcriptional level [77]. Prokop et al. [77] suggested that regulation of prolidase activity is the effect of the crosstalk between IGF-1R and β_1_-integrin receptors through stimulation of ERK1/2 and PI3K/Akt/mTOR pathways by both receptors.

**Figure 3:**
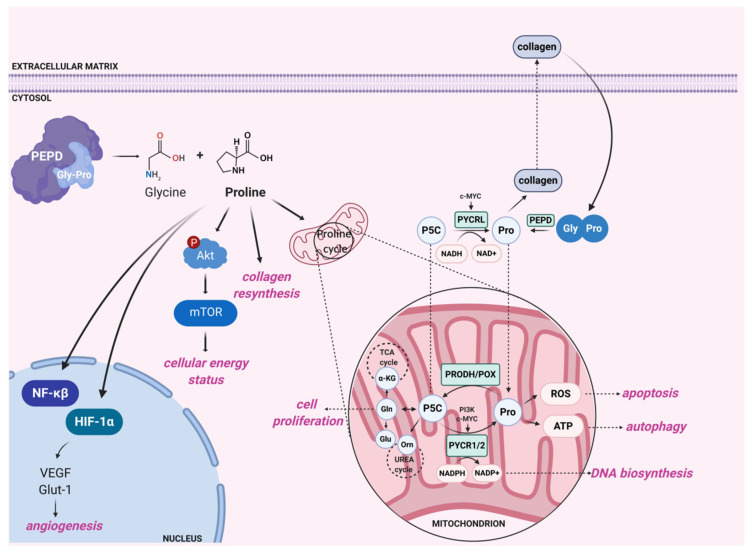
Enzymatic activity of prolidase affects various cellular processes. Prolidase supplies substrates for collagen resynthesis. Proline, the product of prolidase activity, modulates intracellular energetic status via Akt/mTOR pathway, inhibits HIF-1α degradation, and mediates in proline cycle, regulating mitochondrial metabolism. Circled ‘P’ presents phosphorylation event. PEPD—prolidase, Gly-Pro—glycyl-proline, mTOR—mammalian target of rapamycin, Akt—protein kinase B, PI3K—phosphoinositide 3-kinase, HIF-1α—hypoxia-inducible factor 1α, VEGF—vascular endothelial growth factor, Glut-1—glucose transporter 1, NF-κβ—nuclear factor κβ, α-KG—α-ketoglutarate, P5C—pyrroline-5-carboxylic acid, Pro—proline, Gly—glycine, PYCR1/2/L—pyrroline-5-carboxylic acid reductases, Orn—ornithine, Glu—glutamate, Gln—glutamine, PRODH/POX—proline dehydrogenase/proline oxidase, TCA cycle—tricarboxylic acid cycle, ROS—reactive oxygen species, ATP—adenosine triphosphate, NADPH—reduced nicotinamide adenine dinucleotide phosphate, NADP+—nicotinamide adenine dinucleotide phosphate, NADH—reduced nicotinamide adenine dinucleotide, NAD+—nicotinamide adenine dinucleotide. Created with BioRender.com.

In addition to the aforementioned biological processes involving prolidase activity, the enzyme plays a role in the regulation of angiogenesis. Products of prolidase activity, i.e., proline and hydroxyproline, inhibit the degradation of the Hypoxia-inducible factor 1 alpha (HIF-1α) transcription factor via the Von Hippel–Lindau tumor suppressor (VHL)-dependent proteasome pathway. Hydroxyproline acts more potently than proline on the HIF-1α degradation. A possible mechanism explaining this phenomenon is direct or indirect inhibition of proline hydroxylation at positions 402/564 in the oxygen-dependent domain (ODD) of HIF-1α since its hydroxylation is necessary for interaction with VHL [67]. Prolidase is known to affect HIF-1α expression while estrogen receptors are activated [78]. The presence of Pro or Hyp upregulates HIF-1α expression in vitro if cells are cultured with estradiol. The authors observed increased expression of this factor even when estrogen receptors were not stimulated; however, the effect of Pro or Hyp was diminished. It is suggested that α estrogen receptor may connect estrogen-dependent pathways and prolidase activity. HIF-1α-dependent molecules include vascular endothelial growth factor (VEGF), glucose transporter 1 (Glut-1), and TGF-β. They are, respectively, involved in angiogenesis [79], glucose metabolism [80], and control of cell proliferation and differentiation [81]. The study conducted by Surażyński et al. [67] indicated that the level of VEGF and Glut-1 expression is significantly increased under PEPD overexpression. There is evidence that VEGF and Glut-1 overexpression results from the activation of HIF-1α-dependent transcription [67]. VEGF is known to have strong properties for stimulating the expression of α_1_β_1_ and α_2_β_1_ integrin that act as type I collagen receptors in endothelial cells [82], leading to PEPD activity stimulation [73]. Excessive products of prolidase activity stimulate HIF-1α-dependent signaling pathway, boosting the angiogenesis process controlled by VEGF. However, the level of TGF-β expression does not change under PEPD overexpression [67]. Another study indicates that TGF-β and the TGF-β_1_ receptors regulate prolidase activity through proline-dependent signaling. Prolidase activity affects the expression of TGF-β_1_ receptor (TGF-β_1_R) by releasing proline. Prolidase inhibitors (Cbz-Pro, PEP) decrease the expression of this receptor, while, under proline treatment, the expression of TGF-β_1_R is increased. The authors showed that the addition of Pro induces the phosphorylation of kinases from Akt/mTOR pathway, which is associated with cell proliferation and growth [76]. Their results suggest that proline regulates signaling pathways in the cytosol dependent on the TGF-β_1_ receptor. The authors suggest that mTOR is a key element explaining the relationship between prolidase activity and the status of the PI3K/Akt/mTOR pathway. They hypothesize that this kinase acts as a metabolic sensor coordinating the signaling of growth factors, amino acid availability, and cellular energy status. Stimulation of mTOR by phosphorylation promotes pro-proliferative mode of cells, leading to increased protein synthesis with subsequent cell proliferation, growth, and migration [83]. Therefore, proline acts as an intermediary between prolidase activity and regulation of cell survival [76]. Figure 3 shows enzyme-dependent biological processes at the cellular level.

It has also been observed that prolidase activity regulation modulates biological effects of nuclear factor κβ (NF-κβ) transcription factor [65]. Its elevated activity significantly decreases the expression of this transcription factor, which may be related to the increased level of prolidase activity products: Pro or Hyp. It has been evidenced that Pro may protect NF-κβ from activation when the transcription factor is released from the complex with IκBα [84]. Thus, it is likely that high level of Pro or Hyp may prevent IκBα from degradation. NF-κβ is known to strongly inhibit the expression of α_1_ and α_2_ type I collagen subunits [85].

Prolidase activity can also be regulated by non-steroidal anti-inflammatory drugs (NSAIDs). Fibroblasts treated with NSAIDs showed diminished collagen biosynthesis and prolidase activity. It is likely that NSAIDs exhibit inhibitory effect on collagen metabolism by hindering prolidase activity [86]. Similarly to NSAIDs, collagen degradation products inhibit the activity of prolidase [87].

The biological significance of prolidase is to provide free proline as building blocks for collagen resynthesis. Proline may also serve as a signaling molecule and an energy source, or mediate in maintaining redox balance [88]. In recent years, scientists have made more and more efforts to explain the role of proline in reprogrammed energetic metabolism of cancer cells [89]. Proline may come from collagen turnover, but also enzymatic conversion of pyrroline-5-carboxylic acid (P5C), which can be either a precursor or product of proline metabolism. P5C is formed by the enzymatic conversion of glutamate or ornithine by P5C synthase (P5CS) [90] or ornithine aminotransferase (OAT), respectively [91]. P5C reduction occurs due to the activity of P5C reductase (PYCR), while the reaction in the opposite direction is catalyzed by the mitochondrial enzyme—proline dehydrogenase/proline oxidase (PRODH/POX) [92]. The proline cycle plays a crucial role in maintaining the redox balance between the cytosol and mitochondria. P5C acts as a central mediator between the tricarboxylic acid cycle, urea cycle, and proline metabolism [77]. De Ingeniis et al. [93] provided evidence of functional differences between 3 isoenzymes of PYCR (PYCR1, PYCR2, PYCRL). The enzymatic properties of PYCR1 and PYCR2 are similar, while PYCRL exhibits a distinct mode of activity. Because P5C can origin from glutamate or ornithine, these reactions are catalyzed by different P5C reductases. PYCR1 and PYCR2 provide proline from glutamate-derived P5C, while PYCRL reduces P5C from ornithine. The enzymatic conversations are accompanied by distinct cofactors such as reduced nicotinamide adenine dinucleotide phosphate (NADPH) or reduced nicotinamide adenine dinucleotide (NADH). The authors reported that PYCR1 and PYCR2 are mitochondrial enzymes, while PYCRL is present in the cytosol. Elia et al. [94] came to the opposite conclusion, stating that PYCR1 is responsible for the conversion of P5C to proline in the cytosol, which was also suggested by Phang et al. [95]. Proline biosynthesis has been shown to drive the production of the protein necessary for cell proliferation. Pro-proliferative signaling pathways c-MYC and PI3K stimulate the expression of genes associated with proline synthesis (PYCR1, PYCR2, and PYCRL). In addition, c-MYC is known to enhance the conversion of proline from glutamine by stimulating growth and proliferation of cancer cells [96]. The finding that c-MYC increases the expression of enzymes involved in proline synthesis from P5C has shed new light on the understanding of the proline cycle in cancer cells. Silencing PYCRs inhibits tumor growth by reducing the generation of NADP+ and NADPH. As a consequence, their reduced level reduces the supply of nucleotides required for DNA biosynthesis [97]. Intensified proline conversion into glutamine, glutamate, and aspartate promotes cell proliferation. Proline also impacts pentose phosphate pathway that delivers nucleotides for the synthesis of nucleic acids [97,98]. As can be seen in Figure 3, prolidase and proline are responsible for numerous cellular processes. Prolidase activity significantly contributes to proline supply that acts as an intracellular signaling molecule regulating cellular metabolism in many biochemical pathways.

### 3.3. Clinical Significance of Prolidase as a Dipeptidase

The clinical significance of prolidase is reflected in various cancers as well as pathological conditions associated with collagen turnover [4,5,6,7,8,9,10,11,12,13]. For instance, increased prolidase activity has been observed in melanoma [21], breast cancer [22], lung cancer [23], ovary cancer [24], and endometrial cancer [25]. Over the last few years, the structure of PEPD has been studied due to availability of high-throughput techniques. They enabled the detection of several genetic variants of *PEPD* that may be associated with the development of metabolic diseases. No information is available on the pathogenicity of these variants. However, in the literature, there are papers referring to prolidase activity in type 2 diabetes (T2D) and various of its complications (neuropathy, nephropathy, microalbuminuria, diabetic foot). For instance, the genetic variant of *PEPD* (rs3786897) has been identified in the Japanese population to be associated with increased risk of T2D [14]. The same genetic variant was detected in the Chinese population [90]—n-3 fatty acids interacting with this genetic variant modulate the risk of type 2 diabetes. The analysis of a single nucleotide polymorphism (SNP) identified another potentially pathogenic genetic variant. The rs731839 prolidase variant has been associated with affected adiponectin level responsible for insulin resistance and T2D [15]. Another study linked the same genetic variant of prolidase to lipid metabolism [16]. Wu et al. found prolidase variant (rs889140) related to adiponectin metabolism [17], which affects the tissue sensitivity to insulin. All the said genetic variants have not been studied in the context of their pathogenicity. However, there are papers reporting alterations in prolidase activity in metabolic diseases. Patients with T2D accompanied by nephropathic complications show increased plasma prolidase activity (PPA) compared to healthy volunteers [18]. Increased PPA has also been detected in patients with advanced T2D and foot ulcers [19] or co-occurrence of microalbuminuria [20], while in patients with T2D, neuropathy [6] and osteoporosis PPA was reduced [99]. The clinical significance of prolidase activity in these conditions remains unexplained and requires further studies. It is likely that increased activity of prolidase in diabetes and its complications is associated with enhanced collagen breakdown [100]. In contrast, reduced prolidase activity in bone metabolism may result from reduced bone resorption in diabetic patients. In the CNS, the relationship between prolidase and diabetic neuropathy remains unknown. There were attempts to use prolidase activity as an anti-cancer therapy approach. Mittal et al. [21,101,102] synthesized a dipeptide containing C-terminal proline. Since prolidase activity is increased in cancers, the prodrug is released in cancer cells. The results from both in vitro and in vivo experiments are satisfying as they demonstrate inhibited cancer progression. There is available data presenting promising effects of chlorambucil [103] and nitrosoureas [104] linked to L-proline in the MCF-7 breast cancer cell model. However, it is necessary to confirm these results in in vivo models. In summary, numerous literature reports confirm the significant role of prolidase in the clinical aspects associated with both collagen metabolism disorders as well as metabolic and oncological conditions. Further studies need to be conducted to explain the mechanism of prolidase activity in these disorders as T2D or cancers affect more and more people worldwide.

## 4. Concluding Remarks and Future Perspectives

Prolidase is involved in numerous biological processes at the cellular level. PEPD as an enzyme is regulated by signaling pathways dependent on the β_1_-integrin receptor, IGF-1 receptor, and TGF-β_1_ receptor. The catalytic function of PEPD enables the provision of proline or hydroxyproline, which modulate intracellular signaling in the PI3K/Akt/mTOR and ERK1/2 pathways as well as energetic processes. As a result, the cell switches to pro-survival mode promoting DNA synthesis and cell proliferation. In addition, prolidase also has biological properties independent of its enzymatic activity. It plays a regulatory role in the function of other biological molecules. Prolidase is an EGFR and HER2 ligand regulating signaling pathways dependent on these receptors, such as PI3K/Akt/mTOR, ERK1/2, and JAK/STAT3. Under physiological conditions, prolidase stimulates these pathways and may serve as an interface in the regeneration processes under inflammation or tissue damage. Under the overexpression of EGFR and HER2 receptors commonly observed in cancers, the ligand contributes to internalization and lysosomal degradation of these receptors. In the PEPD-p53 complex, prolidase prevents p53 from activation. In plasma, prolidase activates coagulation factors by maintaining its concentration at a normal level. PEPD also participates in immune response by stimulating the expression and maturation of the interferon α/β receptor. Taken together, prolidase could act as a “friend” or “foe” – anti-tumor and pro-tumor enzyme. Promoting EGFR and HER2 degradation seems to be a promising factor in cancer cells, however, prolidase supplying proline stabilizes HIF-1α and, thus, promotes cellular survival in the hypoxic conditions. Similarly, prolidase-PRODH/POX axis could be a crucial mechanism for switching cancer cell mode between ATP (survival) or ROS (apoptosis) generation. Generated ROS can cause DNA damage leading to p53 activation, however, prolidase can protect this transcriptional factor. In the context of cancer milieu, prolidase seems to play an emerging role and its biological activity may be the starting point for further research. Prolidase-based new therapeutic approaches in numerous diseases, including prolidase deficiency, cancers, metabolic disorders, or viral infections may be developed.

## Abbreviations

Akt	Protein kinase B
Ala-Pro	Alanyl-proline
ATM	Mutated in ataxia telangiectasia
ATR	ATM and RAD3-related
BAX	Bcl-2-associated X
BCL-2	B-cell lymphoma 2
cGMP	Cyclic guanosine monophosphate
CHK1	Checkpoint kinase 1
CHK2	Checkpoint kinase 2
EGF	Epidermal growth factor
EP	Enoxaparin
ErbB1/EGFR	Epidermal growth factor receptor
ErbB2/HER2	Epidermal growth factor receptor 2
ErbB3	Epidermal growth factor receptor 3
ECM	Extracellular matrix
FAK	Focal adhesion kinase
Glut-1	Focal adhesion kinase
Gly	Glycine
Gly-Pro	Glycyl-proline
Grb2	Growth factor receptor-bound protein 2
HB-EGF	Heparin-binding EGF-like growth factor
HIF-1α	Hypoxia-inducible factor 1 alpha
Hyp	Hydroxyproline
IFNAR1	Interferon alpha/beta receptor 1
IGF-1R	Insulin-like growth factor 1 receptor
iNOS	Inducible nitric oxide synthase
IκBα	Nuclear factor kappa alpha
JAK	Janus kinase
K_d_	Dissociation constant
Leu-Pro	Leucyl-proline
MAPK/ERK	MAP kinase/Extracellular signal-regulated kinase
MDM2	Murine double minute 2
MDM4	Murine double minute 4
Met-Pro	Methionyl-proline
mTOR	Mammalian target of rapamycin
NADPH	Reduced nicotinamide adenine dinucleotide phosphate
NADP+	Nicotinamide adenine dinucleotide phosphate
NADH	Reduced nicotinamide adenine dinucleotide
NAD+	Nicotinamide adenine dinucleotide
NF-κβ	Nuclear factor kappa beta
NMDA	N-methyl-D-aspartate
NO	Nitric oxide
NS5	Non-structural protein 5
NSAID	Nonsteroidal anti-inflammatory drug
OATT	Ornithine aminotransferase
P5C	Pyrroline-5-carboxylic acid
P5CS	Pyrroline-5-carboxylic acid synthase
PD	Prolidase deficiency
PEPD	Prolidase
Phe-Pro	Phenylalanyl-proline
PI3K	Phosphoinositide 3-kinase
PPA	Plasma prolidase activity
Pro	Proline
PRODH/POX	Proline dehydrogenase/proline oxidase
PYCR1/2/L	Pyrroline-5-carboxylic acid reductase 1/2/L
ROS	Reactive oxygen species
SNP	Single nucleotide polymorphism
Src	Proto-oncogene tyrosine-protein kinase
STAT3	Signal transducer and activator of transcription 3
T2D	Type 2 diabetes
TGF-β	Transforming growth factor beta
TGF-β_1_R	Transforming growth factor beta 1 receptor
Val-Pro	Valyl-proline
VEGF	Vascular endothelial growth factor
VHL	Von Hippel–Lindau tumor suppressor
WIP1	Wild-type p53-induced phosphatase
